# Do Optimal Prognostic Thresholds in Continuous Physiological Variables Really Exist? Analysis of Origin of Apparent Thresholds, with Systematic Review for Peak Oxygen Consumption, Ejection Fraction and BNP

**DOI:** 10.1371/journal.pone.0081699

**Published:** 2014-01-27

**Authors:** Alberto Giannoni, Resham Baruah, Tora Leong, Michaela B. Rehman, Luigi Emilio Pastormerlo, Frank E. Harrell, Andrew J. S. Coats, Darrel P. Francis

**Affiliations:** 1 International Centre for Circulatory Health, National Heart and Lung Institute, Imperial College, London, United Kingdom; 2 Department of Cardiovascular Medicine, Fondazione Toscana G. Monasterio, Pisa, Italy; 3 Norfolk and Norwich Hospital, University of East Anglia, Norwich, United Kingdom; 4 Cardiology Department, Poitiers University Hospital, Poitiers, France; 5 Vanderbilt University School of Medicine, Nashville, Tennessee, United States of America; Scuola Superiore Sant'Anna, Italy

## Abstract

**Background:**

Clinicians are sometimes advised to make decisions using thresholds in measured variables, derived from prognostic studies.

**Objectives:**

We studied why there are conflicting apparently-optimal prognostic thresholds, for example in exercise peak oxygen uptake (pVO_2_), ejection fraction (EF), and Brain Natriuretic Peptide (BNP) in heart failure (HF).

**Data Sources and Eligibility Criteria:**

Studies testing pVO_2_, EF or BNP prognostic thresholds in heart failure, published between 1990 and 2010, listed on Pubmed.

**Methods:**

First, we examined studies testing pVO_2_, EF or BNP prognostic thresholds. Second, we created repeated simulations of 1500 patients to identify whether an apparently-optimal prognostic threshold indicates step change in risk.

**Results:**

33 studies (8946 patients) tested a pVO_2_ threshold. 18 found it prognostically significant: the actual reported threshold ranged widely (10–18 ml/kg/min) but was overwhelmingly controlled by the individual study population's mean pVO_2_ (r = 0.86, p<0.00001). In contrast, the 15 negative publications were testing thresholds 199% further from their means (p = 0.0001). Likewise, of 35 EF studies (10220 patients), the thresholds in the 22 positive reports were strongly determined by study means (r = 0.90, p<0.0001). Similarly, in the 19 positives of 20 BNP studies (9725 patients): r = 0.86 (p<0.0001).

Second, survival simulations *always* discovered a “most significant” threshold, even when there was definitely no step change in mortality. With linear increase in risk, the apparently-optimal threshold was always near the sample mean (r = 0.99, p<0.001).

**Limitations:**

This study cannot report the best threshold for any of these variables; instead it explains how common clinical research procedures routinely produce false thresholds.

**Key Findings:**

First, shifting (and/or disappearance) of an apparently-optimal prognostic threshold is strongly determined by studies' average pVO_2_, EF or BNP. Second, apparently-optimal thresholds always appear, even with no step in prognosis.

**Conclusions:**

Emphatic therapeutic guidance based on thresholds from observational studies may be ill-founded. We should not assume that optimal thresholds, or any thresholds, exist.

## Introduction

Although most clinicians are aware that the majority of biological variables with diagnostic and prognostic value act continuously within populations, they are encouraged to accept recommendations for decision strategies that specify a threshold of a measured continuous variable. Such thresholds often arise from cohort studies that dichotomise patients into subgroups with significantly different prognoses.

Peak oxygen consumption (peak VO_2_) is the most widely accepted quantitative prognostic marker in heart failure following the seminal work of Mancini et al. [1] who reported that cardiac transplantation could be deferred in heart failure patients with a peak VO_2_ of greater than 14 ml/kg/min. Current eligibility for cardiac transplantation, more than twenty years on, still hinges on whether the peak VO_2_ is less than a threshold of 14 ml/kg/min [2] or 12 ml/kg/min in those patients taking beta-blockers [3]. The presence of two conflicting diagnostic thresholds illustrates that studies [4]–[7] and international guidelines [8]–[10] have since assessed a variety of alternative, competing, “optimal” thresholds for peak VO_2_ with conflicting results. Some recent studies even question the prognostic effectiveness of peak VO_2_ [11]–[13], having tested a threshold and failing to find it statistically significant.

The same is true for many other variables used in daily practice. Two examples from imaging and biochemistry, of variables obviously continuous in nature but often dichotomized, are left ventricular ejection fraction (EF)[14]–[16] and Brain Natriuretic Peptide (BNP)[17]–[20]. Each has a range of competing reportedly “optimal” prognostic thresholds.

There are two alternative explanations for these discrepancies. One widely-accepted explanation is that there is a true universal threshold in each variable beyond which prognosis is poor, but modern therapy such as beta-blockade is affecting prognosis so powerfully that the prognostic thresholds have changed [10], [21].

An alternative explanation is that we have misunderstood what a statistically significant difference in prognosis between subgroups tells us. In this explanation, if (for example) a tested peak VO_2_ threshold is far from the middle of a particular cohort, dichotomisation will yield groups of markedly unequal sizes, which would reduce the statistical power to detect a mortality difference between the groups. In contrast, testing a peak VO_2_ threshold nearer the middle, with more equal group sizes, may yield a statistically significant result. If this second explanation is the true one, then variation in the mean value of peak VO_2_ between studies could be enough to make their apparently optimal prognostic thresholds differ.

In this article we comprehensively explore the cause of the discrepancy between studies in their selected optimum prognostic cut point, first by examining published data and separately by performing numerical simulations in which we could know the underlying shape of the relationship between risk factor and risk.

## Methods

### Part 1: Examination of Published Studies

We performed a PubMed literature search (http://www.ncbi.nlm.nih.gov/PubMed) for the three variables of interest (peak VO_2_, LVEF and BNP), in the setting of heart failure, in the period 1990 to 2010. We used as keywords (limit of research: human, all adults 19+ years) “oxygen consumption, heart failure, mortality”, which extracted 287 articles, “ejection fraction, heart failure, mortality”, which extracted 2296 articles, and “BNP, heart failure, mortality”, which extracted 346 articles. Three authors read the full articles to extract the data of interest (as shown in Table 1). Reference lists of these articles were also searched for additional articles.

**Table 1:** The 33 studies reporting a positive (white) or negative (grey) statistical significance of a prognostic threshold of peak VO_2_.

Author	Publication Year	Number of patients	Age (years)	Males (%)	EF (%)	Primary outcome	Max duration of follow-up (months)	Number of events	Mean±SD peak VO2 (ml/kg/min)	Tested threshold (ml/kg/min)	Tested threshold prognostically significant?
Szlachcic	1985	27	56±16	100	22±16	overall mortality	24	14	11,5±1,4	10	yes
Cohn*	1986	273	53±13	100	Nr	overall mortalty	60	nr	15±nr	14,5	yes
Likoff	1987	201	62±10	75	20±10	overall mortality	28	85	13,0±4,0	13	yes
Mancini	1991	116	50±11	84	19±7	overall mortality	25	25	14,7±5,3	14	yes
Parameshwar	1992	127	55±9	89	22±12	overall mortality+urgent transplant	42	41	15,3±5,3	14	yes
Van den Broek	1992	94	57±11	83	22±9	overall mortality	36	21	17,0±5,0	16	yes
Saxon	1993	528	50±12	80	20±7	cardiac mortality+urgent transplant	12	129	12,0±4,0	11	yes
Di Salvo	1995	67	51±10	79	22±7	cardiac mortality	nr	32	11,8±4,2	14	no
Chomsky	1996	185	51±11	78	22±7	cardiac mortality	100	35	12,9±3,0	10	yes
Cohen-Solal	1997	178	52±11	90	25±11	overall mortality+urgent transplant	24	38	17,6±5,6	17	yes
Robbins	1999	470	52±11	71	21±8	cardiac mortality	60	26	18,0±6,0	14	no
Metra	1999	219	55±10	93	22±7	cardiac mortality+urgent transplant	40	29	14,2±4,4	14	yes
Isnard	2000	264	51±12	81	27±10	overall mortality+urgent transplant	82	83	17,1±6,8	14	no
Osman	2000	225	54±12	80	23±13	overall mortality	40	29	16,0±5,9	14	yes
Davies	2000	50	76±5	70	33±14	overall mortality	60	26	15,2±4,5	14,7	yes
Clark	2000	60	59±12	nr	30±15	overall mortality	100	20	19,9±7,7	17,5	yes
Williams	2001	219	56±13	76	Nr	overall mortality	63	27	23,1±9,2	14	no
Ponikowski	2001	80	58±9	76	24±12	overall mortality	36	37	18,3±6,7	14	no
Hansen	2001	311	54±10	84	22±10	cardiac mortality	38	65	14,7±5,5	14	yes
Mejhert	2002	67	74±6	66	36±11	overall mortality	60	14	11,7±3,7	14	no
Gitt	2002	223	63±11	86	29±8	cardiac mortality	24	46	15,8±5,3	14	yes
Rostagno	2003	214	64±10	55	41±14	overall mortality	70	66	18,7±4,1	14	no
Schalcher	2003	146	52±10	87	27±13	overall mortality+urgent transplant	61	41	18,4±5,4	14	no
O' Neill*	2005	1196	54±11	75	19±7	overall mortality	72	nr	16,6±5,1	14	yes
Bard	2006	355	51±10	72	22±8	overall mortality+urgent transplant	46	145	17,3±5,0	14	no
Nanas	2006	98	51±12	89	31±13	cardiac mortality	30	27	19,1±5,9	15	no
Guazzi	2007	288	55±13	67	33±13	cardiac mortality	33	62	15,5±5,0	14,1	no
Guazzi	2007	156	61±9,4	80	35±10	cardiac mortality	42	34	16,8±4,5	14,4	no
Rossi	2007	273	62±9	87	33±8	cardiac mortality	75	40	16,6±4,5	16	yes
Arena	2008	353	59±14	72	28±9	cardiac mortality+urgent transplant	48	104	14,5±5,6	14	no
Kazuhiro	2009	148	63±12	100	35±11	cardiac mortality	67	13	18,2±3,7	14	no
Arena	2010	520	58±12	77	35±14	cardiac mortality	48	79	16,6±6,2	14	no
Sachdeva	2010	1215	53±13	75	23±7	overall mortality	24	234	13,1±2,0	14	yes

EF = left ventricular ejection fraction; nr = not reported.

### Selection criteria

We included all studies on prognostic markers (peak VO_2_, LVEF or BNP) in heart failure that met the following criteria:

quoted a mean or median value for the study population
reported statistical significance of a single threshold

Clinical trials, which might have a confounding effect of allocation to study arms, were excluded, unless they reported results for a control arm independently. We included studies regardless of whether the prognostic threshold was found to be statistically significant or non-significant.

### Part 2: Evaluation in a population known to have no step in risk

We determined, using survival data of a simulated population with a gradual spectrum of a notional continuous risk factor, and definitely no step change in prognosis, whether an “optimal threshold” for the risk factor would appear to arise when the data were analysed by the techniques typically used in prognostic studies and at what value such thresholds appeared.

In the case of peak VO_2_, mortality rises progressively across a wide range, for example giving 2-year mortality of 3%, 7%, 10%, 13%, and 18% in subpopulations with mean peak VO_2_ of 17, 15, 13, 11, 9 ml/kg/min, respectively [22]. For this reason we started simulating a condition in which the relationship between the risk factor and mortality was linear. We subsequently studied non-linear relationships (see below). We deliberately designed the simulation to be applicable to any clinical risk factor.

To do this, we created a simulation of 1500 patients, with a spectrum of a notional risk factor from 0.01 to 15.00, which is linearly related to a patient annual mortality of 0.01–15% (no sharp step in mortality – only a smooth gradation). We simulated using Microsoft Excel survival over 10 years, yielding an ending survival status (alive/dead) and duration for each subject, as required for survival analysis. For example for the 314th patient, whose annual mortality was 3.14%, the survival state was initialized as “alive” and then on 10 occasions (one for each simulated year) he was subjected to 3.14% probability of dying. If the simulated states changed to “dead” in this way, year of death was noted. If he survived all 10 years, the outcome was deemed censored, i.e. “alive” at 10 years.

#### Identifying optimal prognostic threshold by Kaplan-Meier analysis

**Figure 1:**
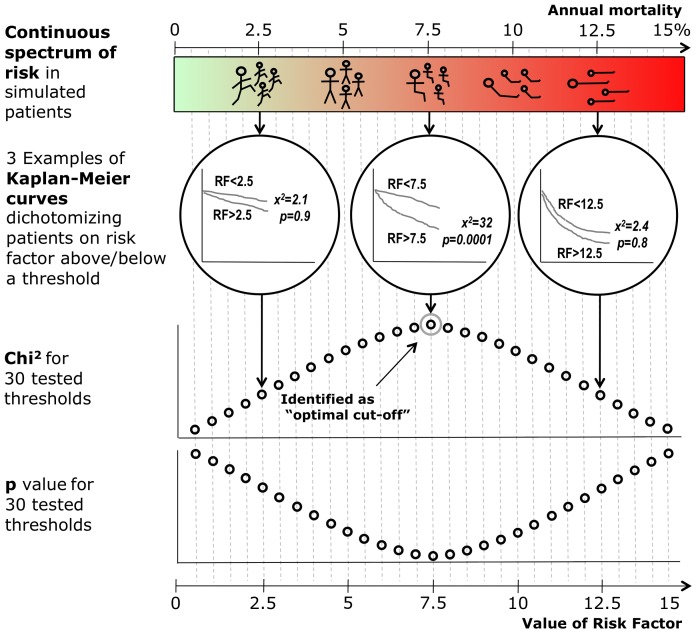
Simulated population characterized by gradually increasing risk and effectiveness of a series of potential prognostic thresholds by Kaplan-Meier and log-rank analysis. In 1500 notional patients, with a wide spread of annual mortality (evenly distributed from 0.01 to 15.00%), we run survival simulation and use Kaplan-Meier and log-rank analysis to examine the prognostic power of many potential threshold values of the risk factor. For three examples amongst the many thresholds tested, the upper panels show the resulting Kaplan-Meier curves. In the lower panels, the results of the full range of tested thresholds are shown. The threshold that gave the highest chi-squared value (equivalent to the smallest p value) was taken as the “optimal” threshold.

We then used Kaplan-Meier analysis to examine the prognostic power of a range of potential threshold values of the risk factor in Statview 5.0 (SAS Institute Inc., Cary, NC). In Figure 1 we show how this was done with three example Kaplan-Meier curves. One threshold is low (2.5), the second is at the median of the group (7.5), and the third is high (12.5). Although only 3 thresholds are shown for illustrative purposes in Figure 1, a wide range of cut-offs were actually tested. In the lower panels, the results of this full range of tested thresholds are shown. The threshold that gave the highest chi-squared value (equivalent to the smallest p value) was taken as the “optimal” threshold.

#### Examining populations of different average risk

To test whether the optimal threshold identified by the procedure described above is a true phenomenon or simply an artefact that tracks the middle of the patients that are studied, we took a series of overlapping 500-patient sub-populations from different parts of the full 1500-patient spectrum and re-ran the analysis within each of these subsets. This mirrors clinical studies examining patient groups with different severities of the disease.

The first such subset covered the lowest risk part of the population spectrum, with the risk factor varying from 0 to 5 and annual mortality accordingly varying from 0 to 5%. The next subset had risk factor 2.5 to 7.5 (annual mortality 2.5 to 7.5%), and so on, until the risk range 10 to 15 (annual mortality 10 to 15%). For each of these subsets, we identified the optimum prognostic threshold of the risk factor by the methods described above.

#### Identifying optimal prognostic threshold by ROC analysis

Separately from the Kaplan-Meier method for identification of the optimal prognostic threshold, we also used ROC analysis to identify the optimal prognostic threshold. We repeated the comparison in each subpopulation with the various subranges of mortality risk as shown above.

#### Identifying optimal prognostic threshold in populations with a non-linear relationship between the variable tested and mortality

In order to extend the applicability of our simulation findings to other risk factors which might not have a simple linear relationship between their value and their associated mortality risk, we repeated the simulation of 1500 notional patients to study different shapes of relationship. We studied a wide range of possible shapes of relationship between risk factor and mortality, including:

- A step (on a background of a linear slope)
- A large step (on a background of a linear slope)
- A step between two plateaus at different levels
- A linear slope segment and then a plateau
- A linear slope segment between two plateaus at different levels
- A plateau segment between two linear slope segments
- A continuously curved relationship (for example, exponential or sigmoidal)

For each possible shape of relationship we ran ten simulations and observed the distribution of apparently-optimal prognostic thresholds in relation to the shape of the relationship between risk factor and mortality.

### Statistical Analysis

Statistical analysis was performed using Statview 5.0 (SAS Institute Inc., Cary, NC). Values are presented as mean±standard deviation (SD) for normally distributed continuous data, as median and interquartile range (IQR) for non-normally distributed continuous data and as percentages for categorical data. p<0.05 was considered statistically significant.

The differences between two groups were evaluated using the Mann-Whitney test and the uncorrected Chi^2^ test, with the highest Chi^2^ being taken as the most statistically significant. Spearman's rank correlation coefficient was used to express the relationship between the apparently-optimal threshold in a group, and the average level of risk factor in that group.

Survival analysis was by the Kaplan-Meier method with the log-rank test.

Apparently-optimal prognostic thresholds were also identified by testing a range of possible thresholds, forming in effect a Receiver-Operating Characteristic (ROC) curve, and then defining as apparently-optimal the threshold that maximised the sum of sensitivity and specificity. To simplify the analysis and minimize problematic right censoring, we designed our simulation to only censor at the end of follow-up.

## Results

### Peak VO_2_ thresholds in published data

Of the 287 studies identified, 113 were excluded because they either had zero or numerous thresholds, 20 because they did not report average peak VO_2_, 29 because they did not report survival, 33 because the setting was not heart failure, and 59 because they were clinical trials with no separate report within the control arm. Therefore 33 studies (8946 patients, Table 1, left hand plot) matched the selection criteria and underwent analysis. Of these, 18 found the threshold in peak VO_2_ [4]–[7], [23]–[35] to be prognostically significant, while 15 found it was not [11]–[13], [36]–[47] (Table 1).

Examining the published studies in cohorts of 5 years from the first published study in 1988, the proportion of studies reporting a statistically significant prognostic threshold for peak VO_2_ has declined from 100% (1986–1990) to 22% (2006–10, p = 0.03 for trend, Table 2).

**Table 2:** Apparent loss of prognostic power of Peak VO_2_ threshold over time and likelihood of different prognostic thresholds giving positive results.

	Number of studies testing a peak VO2 threshold and finding it to be prognostically significant	Number of studies testing a peak VO2 threshold and finding it to be not prognostically significant
**Publication year**		
1986–1990	3 (100%)	0 (0%)
1991–1995	4 (80%)	1 (20%)
1996–2000	6 (75%)	2 (25%)
2001–2005	3 (38)	5 (62%)
2006–2010	2 (22%)	7 (78%)
**Threshold tested (ml/kg/min)**		
9–10.9	2 (100%)	0 (0%)
11–12.9	1 (100%)	0 (0%)
13–14.9	11 (44%)	14 (56%)
15–16.9	2 (66%)	1 (34%)
17–18.9	2 (100%)	0 (0%)

The thresholds chosen for testing varied widely from 10 to 18 ml/kg/min. Studies testing thresholds in the range 13–14.9 and 15–16.9 ml/kg/min were less likely to report positive results (Table 2), and, in particular, studies testing a threshold of 14 ml/kg/min were the *least* likely to be prognostically significant when compared to all the other possible thresholds (44% versus 92%, p = 0.01).

#### Predictors of the peak VO_2_ threshold reported by published studies

**Figure 2:**
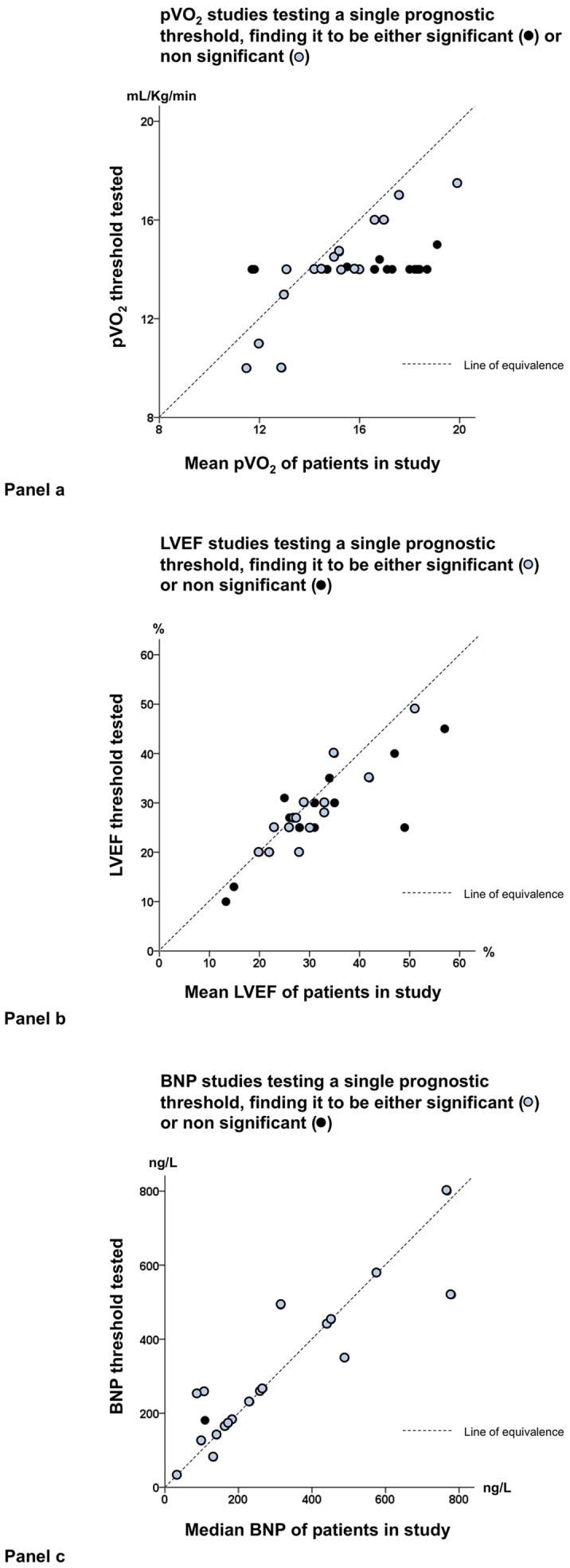
Relationships between the threshold tested and the individual studies' mean: examples from peak VO2 (panel a), LVEF (panel b) and BNP (panel c). In the studies testing a threshold and finding it to be significant (open circles), the threshold reported may be either slightly higher than the mean of the study or slightly lower, but in all cases it is not far from the mean; in contrast it is often far from the mean in the studies testing a threshold and finding it to be non significant (black dots). Dotted lines in each panel represent the line of equivalence.

The variation in optimal peak VO_2_ threshold in the positive studies was almost completely predictable from the individual studies' mean VO_2_ values (r = 0.86, p<0.00001, Figure 2, panel a). There was also a correlation of the threshold with left ventricular ejection fraction (r = 0.60, p = 0.011) and the individual study's mean ejection fraction.

The threshold did not correlate with year of study (r = 0.30, p = 0.23), number of subjects (r = −0.17, p = 0.49), or mean age (r = 0.30, p = 0.23).

#### Why some studies appeared to not confirm a statistically significant prognostic threshold in peak VO_2_

In 15 studies, the peak VO_2_ threshold was found not to be prognostic: Table 3 shows the characteristics of the “positive” versus “negative” studies. The most obvious contender was study size, since larger studies (in the sense of more subjects enrolled, or more subjects with events) would have greater power to detect a threshold. However neither number of subjects, nor number of events, nor any of the main features of the studies or their populations was significantly different between groups.

**Table 3:** Comparison of the main features of studies testing a threshold and finding it to be significant or non significant.

	Studies testing a peak VO2 threshold and finding it to be prognostically significant	Studies testing a peak VO2 threshold and finding it to be not prognostically significant
Sample size (n)	210 (11–282)	214 (98–353)
Mean age (years)	56±6	57±8
Males (%)	83	77
Left ventricular ejection fraction (%)	23.9±4.6	29.8±6.3*
Number of events observed (n)	37 (25–60)	37 (27–79)
Follow-up duration (months)	39 (24–63)	54 (40–64)
Endpoint: cardiac versus all-cause mortality (n)	6/12	8/7
Absolute difference between mean peak VO_2_	1.2±0.8	3.5±2.4**
and threshold tested (ml/kg/min)		

* p<0.01 **p<0.0001 versus studies testing a peak VO_2_ threshold and finding it to be prognostically significant.

Apart from a relatively small difference in ejection fraction (still in the range of severe systolic dysfunction), only one feature differed. The positive studies were all testing thresholds near the individual study means, whereas the negative studies were testing thresholds that were 3 times as far away from the individual study means: absolute difference between VO_2_ threshold tested and mean VO_2_ for the study was 1.2±0.9 ml/kg/min for the positive studies and 3.5±2.0 ml/kg/min for the negative studies, p = 0.0001.

Overall, only five studies also analyzed peak VO_2_ as a continuous variable, four positive studies [24], [25], [27], [30] and one negative study [37]. The negative study [37], was only negative when peak VO_2_ was dichotomized; it confirmed a significant relationship with outcome when peak VO_2_ was analysed as a continuous variable.

### Published thresholds in ejection fraction

Of the 35 studies (out of 2296 studies) matching the inclusion criteria for EF (10220 patients), 22 found the threshold in EF to be prognostically significant [15], [16], [48]–[67], while 13 found it was not (Table 4) [14], [68]–[79].

**Table 4:** The 35 studies reporting a positive (white) or negative (grey) statistical significance of a prognostic threshold of ejection fraction.

Author	Publication Year	Number of patients	Age (years)	Males (%)	Primary outcome	Max duration of follow-up (months)	Number of events	Mean±SD EF (%)	Tested threshold (%)	Tested threshold prognostically significant?
Itoh	1992	298	63±11	57	overall mortality	40	167	35±nr	40	yes
Mehta	1992	112	66±8	74	cardiac mortality	78	31	33±nr	30	yes
Rihal	1994	102	61±14	63	overall mortality	36	35	23±8	25	yes
Omland	1995	145	61±10	80	overall mortality	44	36	51±10	49,1	yes
Andreas	1996	36	54±12	90	overall mortality	53	16	20±8	20	yes
Giannuzzi	1996	508	59±9	88	overall mortality+hospitalization	58	148	26±5	25	yes
Szabo	1997	159	61±nr	85	overall mortality	69	30	26.6±9	27	yes
Anker	1997	171	60±11	90	overall mortality	18	49	30±15	25	yes
Wijbenga	1998	64	59±10	86	overall mortality+transplantation	30	64	31±8	30	yes
Niebauer	1999	99	58±2	90	overall mortality	36	73	13,3±nr	10	no
Metra	1999	219	55±10	93	overall mortality+urgent transplantation	144	38	22±7	20	yes
Isnard	2000	264	51±12	80	cardiac mortality+urgent transplantation	206	83	27±10	27	yes
Ghio	2000	140	52±11	75	cardiac mortality+urgent transplantation	38	52	22±2	20	yes
McDonagh	2001	1640	50,4±nr	48	overall mortality	48	80	47±nr	40	no
Neglia	2002	64	52±12	87	cardiac mortality	82	24	34±10	35	no
Corrà	2002	600	58±7	88	cardiac mortality+urgent transplantation	102	87	26±4	25	yes
Szachniewicz	2003	176	63±nr	86	overall mortality	18	32	42±nr	35	yes
Gardner	2003	142	50±10	82	cardiac mortality+urgent transplantation	55	24	14.9±7	13	no
Martinez-Selles	2003	1065	75±nr	49	overall mortality	51	507	35±7	30	no
Shiba	2004	684	67±13	66	overall mortality	33	175	49±15	25	no
Guazzi	2005	128	60±9	79	cardiac mortality	51	24	34±10	35	yes
Kistorp	2005	195	69±nr	71	overall mortality	30	46	30±8	25	yes
Junger	2005	209	54±10	86	overall mortality	35	45	22±10	20	yes
Peterson	2005	61	53±11	86	overall mortality+urgent transplantation	139	32	26±9	27	no
Bloomfield	2006	549	56±10	71	cardiac mortality	24	51	25±6	31	no
Rossi	2007	273	62±nr	87	overall mortality	45	44	31±3	30	yes
Arslan	2007	43	62±10	86	overall mortality	24	16	35±6	30	yes
Guazzi	2007	288	55±13	62	overall mortality	33	62	33±13	28	yes
vonHaeling	2007	525	61±12	94	overall mortality	28	171	28±4	20	yes
Nishio	2007	145	67±1.8	70	cardiac mortality+hospitalization	33	28	31±nr	30	no
Dini	2007	356	70±6	22	cardiac death	34	54	31±3	25	no
Whalley	2008	228	70±3	66	cardiac mortality+hospitalization	18	26	57±12	45	no
Dini	2008	142	71±11	78	overall mortality	50	85	28±7	25	no
Parissis	2009	300	65±11	83	cardiac mortality+hospitalization	12	92	28±4	25	no
Smilde	2009	90	60±8	85	overall mortality	156	47	29±9	30	yes

EF = left ventricular ejection fraction; nr = not reported.

In the 22 studies where EF was found to be prognostically significant, the threshold varied widely from 20 to 49%, but was strongly associated with study sample means (r = 0.90, p<0.0001, Figure 2, panel b). In contrast, in the 13 studies where EF was found to be not prognostically significant, the tested threshold was relatively far (124% further than positive studies) from the individual study means: absolute difference between EF threshold tested and mean EF for the positive study averaged 2.5±2.3% for the positive studies and 5.8±6.5% for the negative studies, p<0.05). Examining the published studies in cohorts of 5 years from the first published study in 1992, again a progressive decline was observed in the percentage of studies reporting a threshold which was prognostically significant, from 100% (1991–1995) to 45% (2006–2010).

### Published thresholds in Brain Natriuretic Peptide

Of 20 studies (out of 346 studies) matching the inclusion criteria for BNP (9725 patients), 19 studies found the threshold in BNP to be prognostically significant [17], [20], [80]–[95], and one study found it was not (Table 5) [96]. In the positive studies, the threshold widely varied from 132 to 800 ng/L, but was again strongly determined by the study median (r = 0.86, p<0.0001, Figure 2, panel c).

**Table 5:** The 20 studies reporting a positive (white) or negative (grey) statistical significance of a prognostic threshold of brain natriuretic peptide.

Author	Publication Year	Number of patients	Age (years)	Males (%)	Primary outcome	Max duration of follow-up (months)	Number of events	Median (IQR) BNP (ng/L)	Tested threshold (ng/L)	Tested threshold prognostically significant?
Omland	1996	131	68±1	75	overall mortality	48	31	33,1 (nr)	33,3	yes
Yu	1999	91	61±nr	70	cardiac mortality	12	25	165 (nr)	165	yes
Bettencourt	2004	84	69±9	60	overall mortality	nr	17	260,4 (122,4–543,8)	260,4	yes
de Groote	2004	150	55±13	nr	cardiac mortality	24	35	107 (3,5–876)	260	yes
Hulsmann	2005	112	68±12	64	overall mortality	43	nr	231 (nr)	231	yes
Watanabe	2005	417	64±14	69	overall mortality+hospitalization	nr	124	132 (nr)	81	yes
Lamblin	2005	546	56±nr	82	cardiac mortality+urgent trasplantantion	53	113	173 (nr)	173	yes
Bertinchant	2005	63	54±7.2	89	cardiac mortality+hospitalization	nr	47	89,5 (11–1413)	254	yes
Horwich	2006	316	53±13	74	overall mortality	48	nr	452 (nr)	452	yes
Masson	2006	3916	nr	nr	overall mortality	nr	758	99 (nr)	125	yes
Sun	2007	50	67±6	58	cardiac mortality	24	12	780 (nr)	520	yes
Frantz	2007	206	60±nr	80	overall mortality+hospitalization	12	81	141 (nr)	141	yes
Christ	2007	123	63±12	85	overall mortality+urgent trasplantantion	36	28	183 (11–1672)	183	yes
Dhaliwal	2009	464	67±7	99	overall mortality+hospitalization	nr	126	490 (233–796)	350	yes
Moertl	2009	96	69±12	58	overall mortality+hospitalization	24	34	267 (nr)	267	yes
Niessner	2009	351	75±nr	66	overall mortality+hospitalization	16	175	441 (231–842)	441	yes
Cohen-Solal	2009	1038	66±nr	70	overall mortality	6	nr	768 (nr)	800	yes
El-Saed	2009	173	67±11	98	overall mortality	24	31	315 (nr)	492	yes
Voors	2009	224	68±10	70	overall mortality+resuscitated arrest	12	63	109 (nr)	181	no
Sachdeva	2010	1215	53±13	75	overall mortality+urgent trasplantantion	24	442	575 (190–1300)	579	yes

EF = left ventricular ejection fraction; nr = not reported.

### Survival simulation study

#### Thresholds from Kaplan Meyer analysis

**Figure 3:**
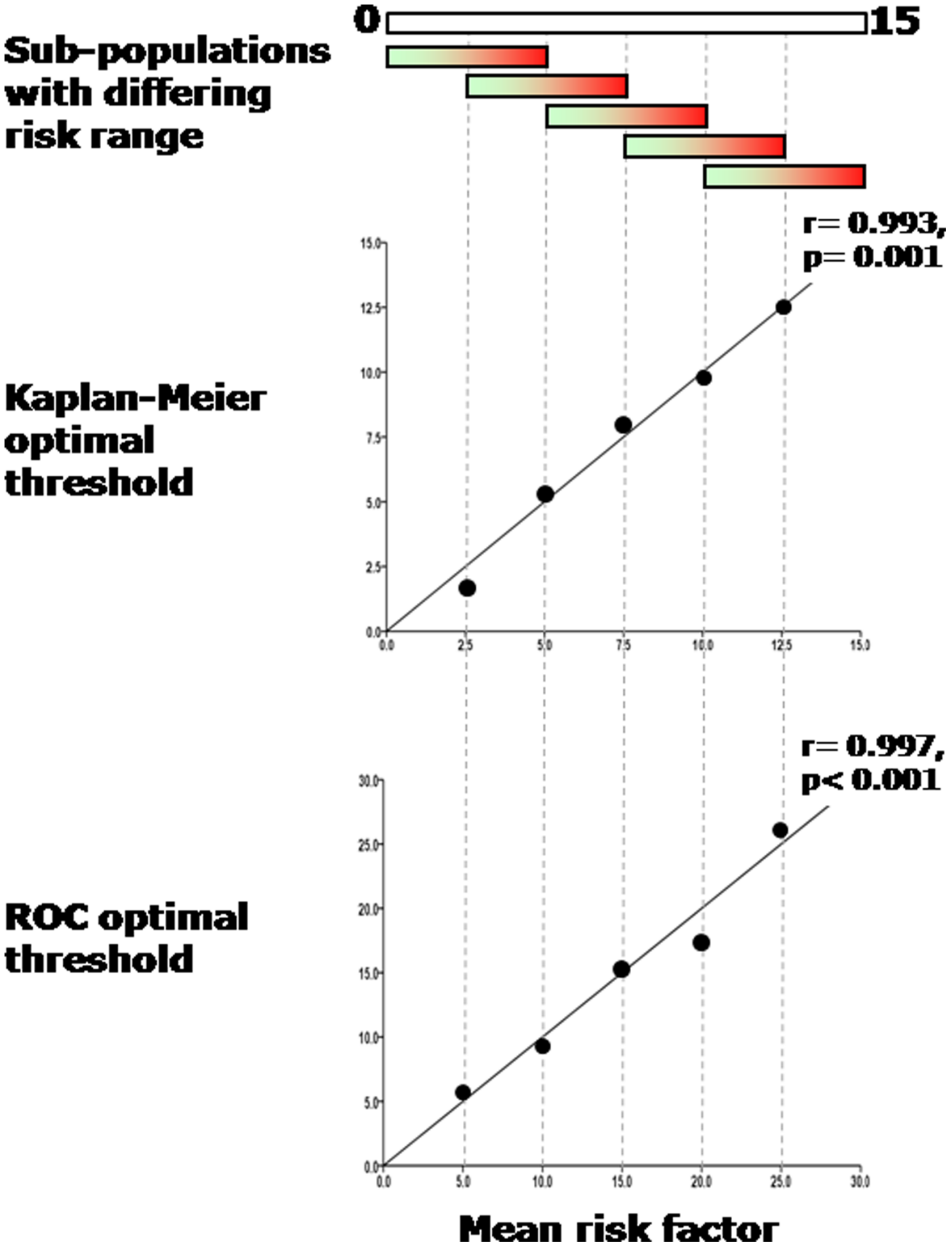
Mathematical simulation of sample selection from the general population: correlations between the sample mean and the apparently-optimal prognostic threshold. Sub-populations with different ranges of risk simulating a shift in the mean peak VO_2_ were created and strong correlations between population mean and optimal thresholds by Kaplan-Meier and ROC analysis were found.

**Figure 4:**
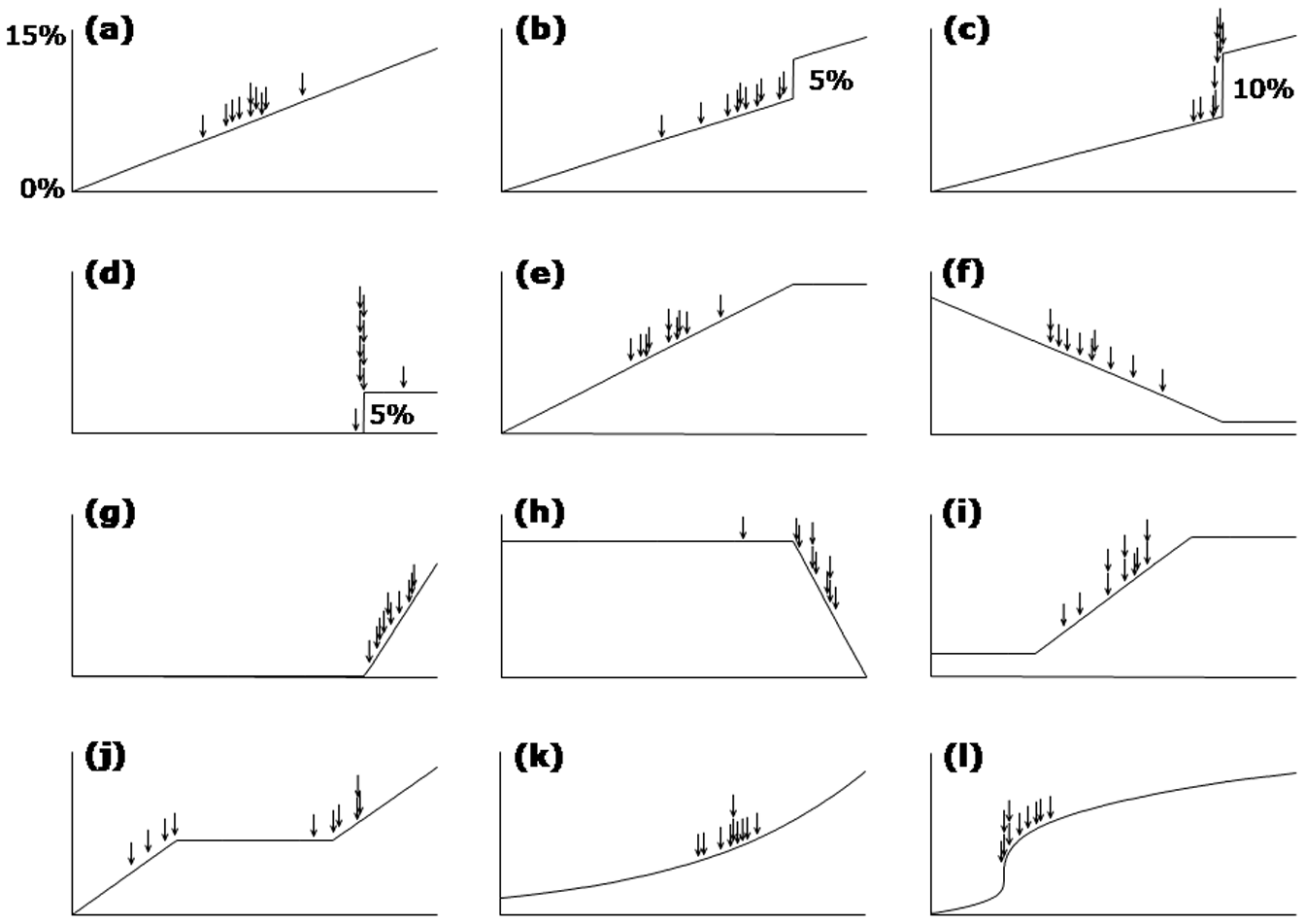
Apparently-optimal prognostic thresholds in twelve different types of relationship between the risk factor and mortality. For each type of relationship, 10 simulations were conducted, and the 10 apparently-optimal thresholds derived from Kaplan Mayer analysis were found. They are shown by vertical arrows (where multiple arrows would have been superimposed, they have been placed one above another).

**Figure 5:**
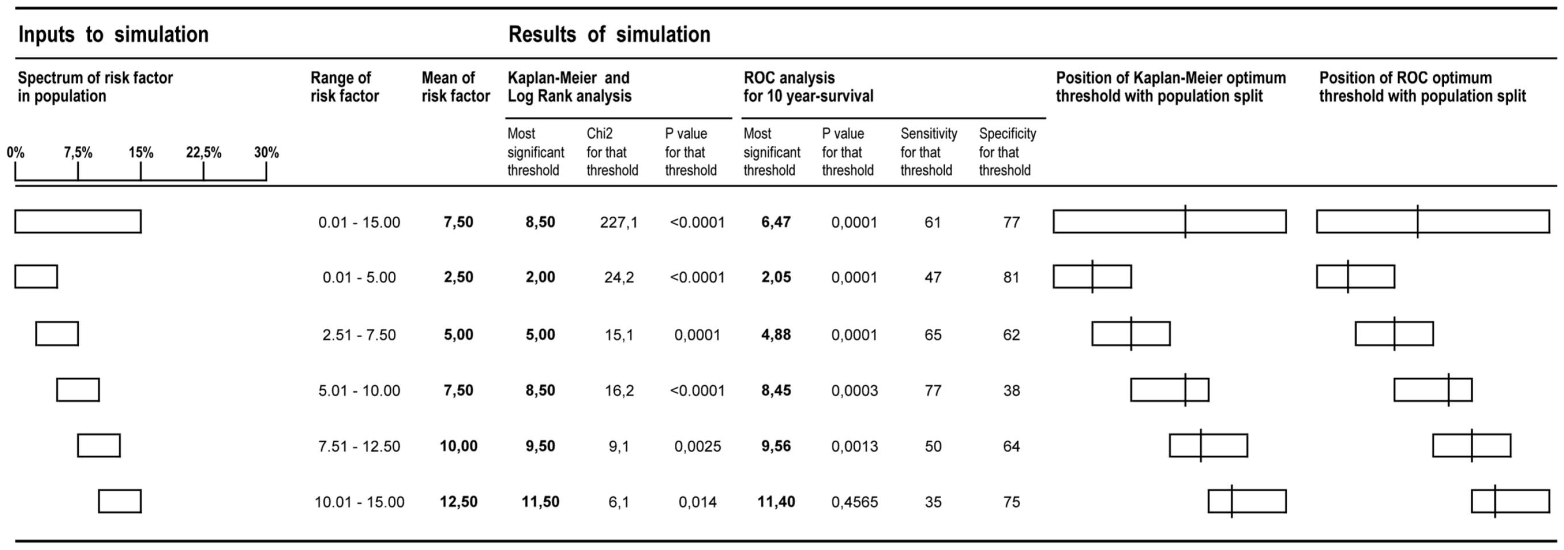
Apparent optimal prognostic threshold, by Kaplan-Meier and ROC method, arising from a mathematically simulated population with known, smooth gradation of risk. The position of the apparently optimal threshold is almost completely determined by the risk factor mean. Several overlapping samples are taken from a single population of smoothly varying risk.

In these simulations, even with a purely smooth gradation of risk and definitely no step change, each 1500-patient population yielded its own apparent “optimal” prognostic threshold (Figure 3, Figure 4 panel a and Figure 5).

This apparent optimal threshold was always close to the mean of the population being studied, because in general thresholds tested far from the mean consistently had lower prognostic power. As we moved across the spectrum of risk examining different sub-populations of 500 patients with different average risks, drawn from the main population, we observed an almost exactly corresponding change in the optimal threshold as calculated by the Kaplan-Meier method (Figure 5). This was true for each sub-population tested (with samples characterized by an annual mortality of 0–5%, 2.5–7.5%, 5–10%, 7.5–12.5, 10–15%, Figure 5). We observed a strong correlation between the optimal threshold within a population and the mean risk factor within that sub-population (r = 0.99, p<0.001 Figure 3).

#### Thresholds from ROC analysis

The ROC analysis, like the Kaplan-Meier analysis, also found an apparently optimal prognostic threshold in each simulated population even though they definitely had only smoothly-varying risk. Again, this apparently-optimal threshold in the risk factor was found to shift to match the average risk factor level in the patient subset (r = 0.99, p<0.001, Figure 3, Figure 5).

#### Identifying optimal prognostic threshold in populations with a non linear relationship between the variable tested and mortality

When we employed a nonlinear relationship between risk factor and mortality, some subtleties emerged. If the risk factor was linearly predictive of mortality, then the apparent optimal prognostic threshold was found to be simply approximately the middle of the population (Figure 4, panel a). If there was a step increase in mortality on a background of an approximately linear gradation, the step was reliably identified as long as it was distinctly larger than the gradation (Figure 4, panels b and c). If the risk factor was simply a step relation with mortality, with no gradation above or below that step, then that step was found, even if small (Figure 4, panel d).

If there was a slope of risk and a plateau (as is likely with some real-life risk factors such as peak VO_2_, EF and BNP) the location of the apparently optimal threshold was more complex. In situations where most of the patients were on the plateau, then the optimal threshold lay at the junction between plateau and gradient. If, on the other hand, most of the patients were on the gradient, then the apparent optimal threshold lay about half-way along the gradient (Figure 4, panels e, f, g and h). These latter two observations were true regardless of whether it is a rising or falling gradient.

If the risk shape was, instead, a slope between two plateaus, the middle of the slope was the most favoured location for the apparently optimal threshold (Figure 4, panels i). If there was a plateau between two slopes, the optimal threshold tended to be near the end of (either) one of the slopes, where it meets the plateau (Figure 4, panel j). If there was a smooth curve of mortality (regardless of whether convex or concave) the apparent optimal threshold lay near the middle, but a little displaced toward the steeper side of the curve (Figure 4, panels k and l).

## Discussion

In this study we have identified using the most commonly used prognostic measurements in heart failure, namely peak VO_2_, EF and BNP, that commonly-used methods of defining an apparently “optimal” prognostic threshold can be simply a manifestation of the middle of the risk factor spectrum of the individual population studied, and should never be taken to signify any meaningful step change in prognosis. Even in an artificial population known to consist of a completely smooth gradation of risk, such methods give an apparent prognostic threshold but its location reflects little more than the population average.

### Does the finding of a clear optimal threshold with Kaplan-Meier analysis mean that there is really a step change in prognosis?

We deliberately simulated notional populations without step increase in risk but rather gradually increasing risk, and examined the effectiveness of a series of potential prognostic thresholds. The most significant difference between the Kaplan-Meier curves was found when the threshold was near the mean population risk. As the tested threshold was moved progressively further from the middle of the population in either direction, the Kaplan-Meier curves became less statistically significantly separated, so that dichotomising near the extremes of low or high values of risk cause the curves to be not statistically significantly different from each other.

The commonly-used methods produce an apparently-optimal prognostic dichotomy point effortlessly, but there is no real clinical phenomenon occurring at that point. Maximally-significant separation of the Kaplan-Meier curves need not represent a biological step change: it could easily be merely identifying the middle of that risk factor in that individual study, in a manner that is opaque, expensive and roundabout.

### Does ROC analysis resolve the pitfalls of the Kaplan-Meier approach to finding a biological threshold?

ROC analysis has a reputation for making statistical analysis of diagnostic value more comprehensive. It has been used in some studies to identify an optimal threshold of peak VO_2_ [97]–[99].

However, our simulated populations show that ROC analysis is as susceptible as the Kaplan-Meier method, i.e. it tends to find the optimal threshold to be the middle of the population.

Neither Kaplan-Meier nor ROC methods can be relied upon to be illuminating a true biological threshold in prognosis. Each is heavily biased towards reporting the centre of the risk spectrum of that study. Indeed, the search for such dichotomies has been demonstrated to be a seriously underpowered way to look for prognostic relationships [100].

### Lessons learnt from peak VO_2_, EF, and BNP studies

Paradoxically, while early studies were unanimous in confirming particular threshold values of peak VO_2_ to be prognostically important in heart failure [4]–[6], [23]–[25], more recent studies seemed to cast doubt on this, with only a quarter of studies between 2003–2010 confirming statistically significant prognostic cut-off values. Further, the widely recommended threshold of 14 ml/kg/min [8]–[10] was found to be the *least* likely be statistically significant.

The explanation for this appears to be that the significant, and in general older, studies tested several values and picked the most significant (or deliberately used the middle of their population), benefitting from the flexibility to choose their own threshold, close to their mean peak VO_2_. The studies that found no prognostic relationship, which tended to be more recent, chose to test the clinically established threshold of 14 ml/kg/min as their cut-off value, which happened to be relatively far away from their own population mean.

A similar pattern was seen with EF. The community is aware that for EF there is no special universal prognostic threshold and even clinical guidelines [101] recognise that a sharp change in prognosis at a threshold is unlikely.

BNP is a more recent entrant. 95% of studies found BNP to be prognostic, which may be a sign of its strong prognostic value, or the relative ease of conducting large studies, or the lack of a rigid predetermined threshold to test against. Even up to 2005, guidelines resisted the temptation to specify a prognostic threshold for BNP [102], and by 2008 when pressure for a diagnostic threshold became irresistible, this was kept 300% wide (100–400 pg/ml), perhaps subtly telegraphing the undesirability of a threshold out of context of clinical background information and individual risk-benefit evaluation [103].

Selecting “optimal” cut points without a strong reason to suspect a true biologic threshold is unwise [104]–[106]. It may better to assume a smooth graded relationship of a continuous variable with outcome. Moreover, excessive reverence for a statistically optimal single cut point and cementing of it in clinical guidelines, may impair that variable's prognostic power when compared with other variables proposed later. Taken to its extreme, setting cut points that are effectively the middle of the first positive study can lead to artificial discovery of new prognostic markers statistically independent of the old (because the old are handicapped).

### Two easily-confused but different types of “threshold”

It is important to distinguish between two different entities, each of which might reasonably be called a “threshold”. The first, discussed extensively in this study, is the value of a variable which most impressively separates a population into high-risk and low risk groups: an “observed prognostic threshold”. This study shows that such observed thresholds routinely arise even when the variable has a non-stepped, smoothly continuous relation to risk. A better term than “optimal risk threshold” would be “middle of the risk spectrum”, albeit less exciting.

The second type of threshold is the “clinical decision-making threshold” which is more subtle. Physicians need at times to decide whether to intervene: this is a dichotomy with no intermediate status. Correct decision-making depends on comparing the risk of intervening against the risk of not intervening, in the context of how the individual patient views such risks. Only in an imaginary disease with somehow just one important variable, and in which patients consistently value outcomes in the same way as a statistical model does, might a decisional threshold be applicable. Even still, this would be different from identifying a step change in prognosis, and certainly different from identifying the most statistically significant breakpoint (often simply the middle of the studied group).

That these two types of threshold differ is sketched in Figure 5, which imagines a situation where, with only medical therapy, mortality falls smoothly with rising peak VO_2_, while with transplantation mortality is at a fixed level. In this thought experiment, it is assumed that no other variables are relevant. Above a certain level of peak VO_2_, medical therapy is safer; below it, transplantation is safer. This is therefore the ideal clinical-decision-making threshold. But if improved medical therapy were developed, for example, this ideal decision-making threshold moves left. Exactly where this decision-making threshold lies cannot established by looking only at outcomes in non-transplanted (or transplanted) population alone. It can only be established by examining outcomes in both non-transplanted and transplanted populations. In real life, other variables are very important, and therefore the decision-making threshold cannot be established by comparing outcomes in patients who have been allocated by routine clinical methods to transplant or no transplant. A randomized controlled trial is the most secure basis, because this design gives the best chance of matching all variables, both those that can be observed and quantified and those that cannot.

### Prognostic studies

**Figure 6:**
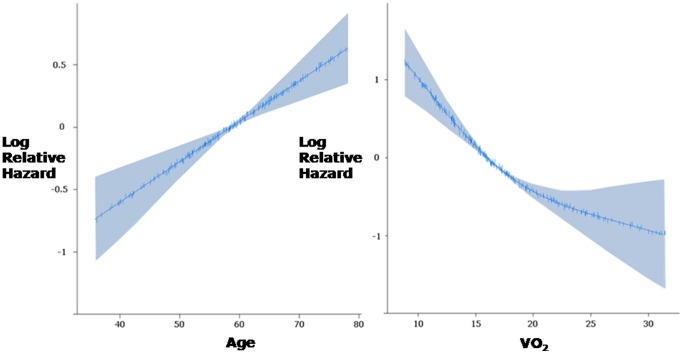
Two different types of threshold: apparently-optimal versus decision-making thresholds. Cartoon illustrating two distinct, unrelated, values that are both called “threshold”. The statistically optimal threshold value of a continuous risk factor for subdividing the population (left panel) has no relevance to the question of what value of a risk factor should be used to decide whether to intervene or not (right panel). The former, the “observed prognostic threshold”, will generally be the middle of whatever population happens to be studied, if mortality varies roughly linearly with the risk factor. The latter, the “ideal clinical decision-making threshold”, will critically depend also on the outcomes with intervention, and will move as the success of the package of medical therapy (and of transplantation) changes with time. There is no sense in using one as a proxy for the other.

If it is desired to test for a prognostic threshold in a variable, there are straightforward statistical methods for doing so. For example, a flexible nonlinear function can be fitted and displayed with confidence bands for incremental log odds over the whole span of the marker; seeking a point such that risk is flat on both sides of that point but the risk on one side is much different from the risk on the other side (Figure 6). Such a phenomenon amongst cardiovascular prognostic studies is a rarity.

**Figure 7:**
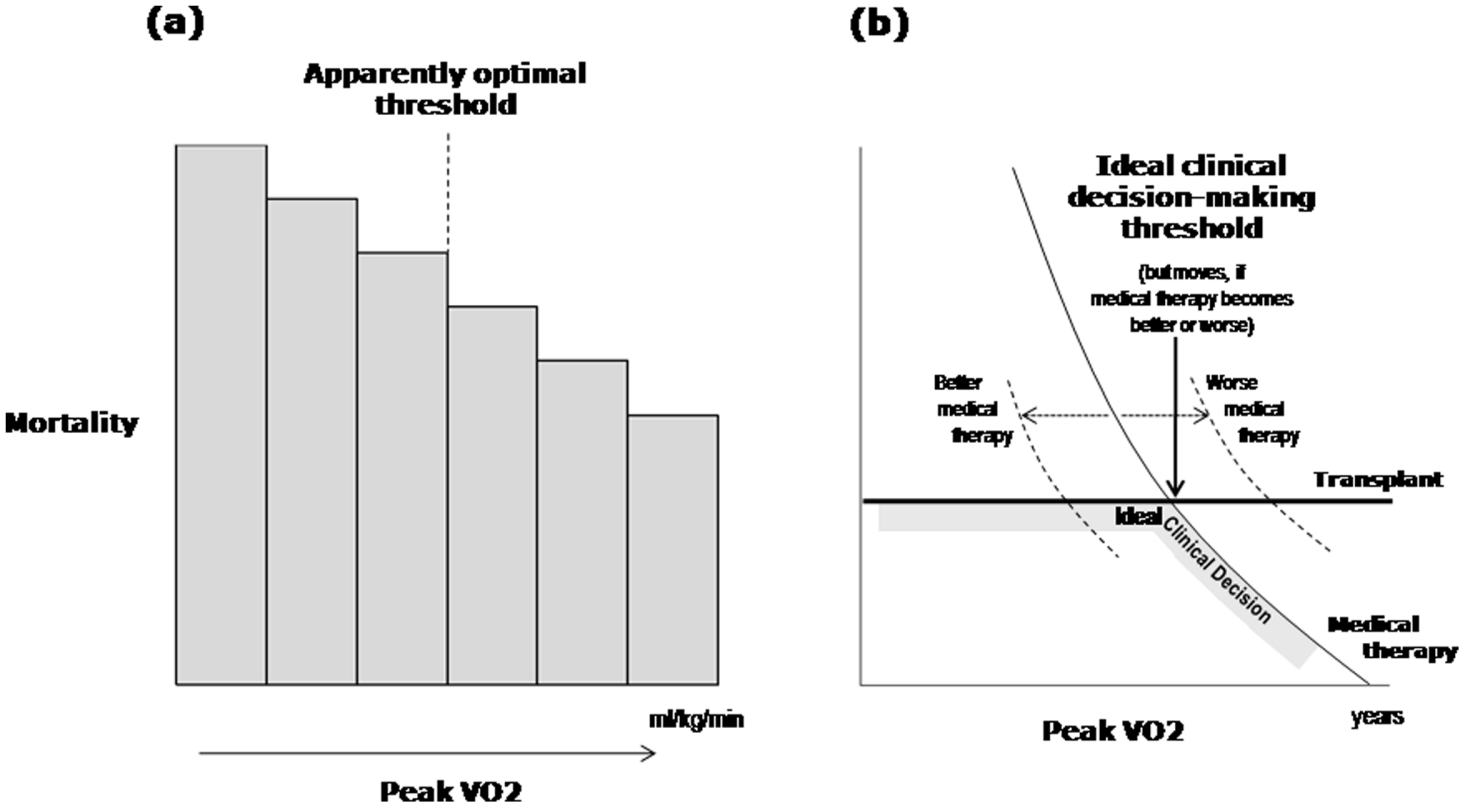
Example of use of flexible non-linear function to describe the relationships between age (left) and peak VO_2_ (right) and log odds of death using 208 patients. The shaded areas represent the 95% confidence intervals for this function. Flexible non-linear functions have numerous benefits over categorization, including improved precision, avoidance of assumption of a discontinuous relationship, maximisation of applicability to the individual and importantly avoidance of giving other variables or interactions artificially high weights. Inspection of the resulting plots above can make obvious the lack of a discontinuity in risk.

If for academic reasons there is a desire to seek a clinical decision-making threshold for a condition that has a single dominant prognostic marker, the reliable method is to conduct a randomized controlled trial which enrolls patients with values in the vicinity of the suspected threshold, and see where (with random allocation) the flexible nonlinear risk curves cross over (Figure 7). For all diseases evaluated by continuously distributed variables, the location of this crossover will always have a wide uncertainty (error bar) unless a very large number of events occur. Pooled analysis using multiple trial datasets has successfully used this approach to explore a decision-making threshold in QRS duration for implantation of biventricular pacing devices [107].

Without elucidation of why we believe thresholds exist it might be difficult to advance our methods of deciding on advanced intervention (such as transplantation, or device implantation) beyond their current state. Continuous markers such as peak VO_2_, EF and BNP can be treated alongside other risk markers in multivariate fashion to finely grade prognosis. Clinging to or arguing over particular historically-documented threshold values may impede, rather than support, advances such as incorporating new information from potentially simple, cheap and effective supplemental prognostic markers [108]–[110]. Simple clinical variables such as age, sex and ECG QRS duration may capture as much or more prognostic power as more elaborately-obtained variables [108], [111]. Even strong markers when used in this dichotomous fashion may not live up to expectations [112]. Recognising and displaying [113] their continuous and progressive value may be preferable [114]. Cutpoints can synthesise apparent relationships when there are really none [115], and apparently-optimal diagnostic cutpoints can shift substantially with change in even a simple covariate such as cough [116].

Nor is it correct to assume that maximisation of diagnostic accuracy is a wise target, since this is only optimal if false positive and false negative categorisation are exactly equally undesirable. Cutpoints, especially when automatically constructed, impede our ability to understand the spectrum of risk, hide the existence of the intermediate zone, and encourage information destruction.

### Clinical implications

Reporting an optimal prognostic threshold of a variable, without enumerating the actual shape of the risk profile, may be little more than an elaborate and time-consuming way of describing the middle of the population being studied. Conversely studies testing a pre-specified prognostic threshold, and finding no statistical significance, do not invalidate the prognostic meaning of the variable, especially if the average value in that study is far from the pre-specified threshold.

When making decisions about individual patients in the clinical setting we as physicians are often cautious about extrapolating from studies, acknowledging the differences between the population recruited (and the care delivered) in formally designed trials versus “real-life” practice. This same caution is rarely extended to the application of cutpoints to the individual patient, even though published cutpoints turn out to often be merely an indirect index of the middle of the sample described. We therefore risk treating patients simply according to whether, in the context of a previous study, they are above-average or below-average.

It might well be reasonable for a resource in short supply to be offered to simply the higher risk half of the population, but we should openly state that the threshold for therapy is merely the mid-point of the first adequately-powered prognostic study; it is not necessary to pretend that a threshold identified thus has any physiological universality or clinical permanence. This applies not only to heart failure but throughout clinical medicine, since many prognostic variables (e.g. blood pressure, cholesterol, prostate specific antigen) are continuous variables.

Clinician scientists wishing to ascribe special status to a threshold should perhaps be obligated to provide evidence of several criteria.

- There must be a difference in outcome below versus above the threshold.
- There should be almost flat risk profiles on both sides of the threshold.
- Enough data should be accrued to test whether the threshold is a true point of discontinuity when risk is evaluated using a flexible function of the marker.

For commonly-used cardiological markers, the second and third will only rarely be confirmed.

### Study limitations

This study does not prove the cause of the disagreement in optimal threshold in peak VO_2_ or EF or BNP between studies, or of the apparent loss of prognostic significance of this parameter over time. It only shows that the most statistically significant threshold has nothing to do with the optimal clinical decision-making threshold, nor is its existence evidence of any specialchange in risk at that point.

This study cannot establish the optimal clinical decision-making thresholds for therapy. If they exist, they can only be obtained reliably by randomized controlled trials.

## Conclusions

Conflict between reported optimal prognostic thresholds in variables such as peak VO_2_, EF, BNP between studies result almost entirely from differences in average values of these variables between studies.

Clinical guideline writers should hesitate to specify a threshold in a variable for therapeutic decisions arising from such observational studies. Their readers might question how a committee can know what is best for an individual patient whom it has not met, knowing only whether one continuous variable is above or below an essentially meaningless threshold; this might weaken the credibility of the guideline as a whole.

Manuscript authors should not expend effort synthesising, and clinicians should not spend time reading, unnecessarily elaborate explanations for apparent movement of thresholds between studies, since the widely-used procedures generate for almost any continuous risk factor an artifactual apparently-optimal threshold near the middle of any patient group examined. We should study prognosis without these misapprehensions.

## Supporting Information

Checklist S1 **PRISMA checklist.**
(DOC)
