# Correction: Do Optimal Prognostic Thresholds in Continuous Physiological Variables Really Exist? Analysis of Origin of Apparent Thresholds, with Systematic Review for Peak Oxygen Consumption, Ejection Fraction and BNP

**DOI:** 10.1371/journal.pone.0105175

**Published:** 2014-09-17

**Authors:** The PLOS ONE Staff

Tables 1, 4, and 5 are incorrectly shaded, and the shading does not denote negative statistical significance. The correct versions of Tables 1, 4, and 5 can be viewed below, with lines typeset in boldface indicating negative statistical significance.

**Table 1:** The 33 studies reporting a positive or negative statistical significance of a prognostic threshold of peak VO_2_.

Author	Publication Year	Number of Patients	Age (years)	Males (%)	EF (%)	Primary outcome	Max duration of followup (months)	Number of Events	Mean± SD peak VO2 (ml/kg/min	Tested threshold (ml/kg/min)	Tested threshold prognostically significant?
Szlachcic	1985	27	56±16	100	22±16	overall mortality	24	14	11,5±1,4	10	yes
Cohn*	1986	273	53±13	100	nr	overall mortalty	60	nr	15±nr	14.5	yes
Likoff	1987	201	62±10	75	20±10	overall mortality	28	85	13,0±4,0	13	yes
Mancini	1991	116	50±11	84	19±7	overall mortality	25	25	14,7±5,3	14	yes
Parameshwar	1992	127	55±9	89	22±12	overall mortality + urgent transplant	42	41	15,3±5,3	14	yes
Van den Broek	1992	94	57±11	83	22±9	overall mortality	36	21	17,0±5,0	16	yes
Saxon	1993	528	50±12	80	20±7	cardiac mortality + urgent transplant	12	129	12,0±4,0	11	yes
**Di Salvo**	**1995**	**67**	**51±10**	**79**	**22±7**	**cardiac mortality**	**nr**	**32**	**11,8±4,2**	**14**	**no**
Chomsky	1996	185	51±11	78	22±7	cardiac mortality	100	35	12,9±3,0	10	yes
Cohen-Solal	1997	178	52±11	90	25±11	overall mortality + urgent transplant	24	38	17,6±5,6	17	yes
**Robbins**	**1999**	**470**	**52±11**	**71**	**21±8**	**cardiac mortality**	**60**	**26**	**18,0±6,0**	**14**	**no**
Metra	1999	219	55±10	93	22±7	cardiac mortality + urgent transplant	40	29	14,2±4,4	14	yes
**Isnard**	**2000**	**264**	**51±12**	**81**	**27±10**	**overall mortality + urgent transplant**	**82**	**83**	**17,1±6,8**	**14**	**no**
Osman	2000	225	54±12	80	23±13	overall mortality	40	29	16,0±5,9	14	yes
Davies	2000	50	76±5	70	33±14	overall mortality	60	26	15,2±4,5	14.7	yes
Clark	2000	60	59±12	nr	30±15	overall mortality	100	20	19,9±7,7	17.5	yes
**Williams**	**2001**	**219**	**56±13**	**76**	nr	**overall mortality**	**63**	**27**	**23,1±9,2**	**14**	**no**
**Ponikowski**	**2001**	**80**	**58±9**	**76**	**24±12**	**overall mortality**	**36**	**37**	**18,3±6,7**	**14**	**no**
Hansen	2001	311	54±10	84	22±10	cardiac mortality	38	65	14,7±5,5	14	yes
**Mejhert**	**2002**	**67**	**74±6**	**66**	**36±11**	**overall mortality**	**60**	**14**	**11,7±3,7**	**14**	**no**
Gitt	2002	223	63±11	86	29±8	cardiac mortality	24	46	15,8±5,3	14	yes
**Rostagno**	**2003**	**214**	**64±10**	**55**	**41±14**	**overall mortality**	**70**	**66**	**18,7±4,1**	**14**	**no**
**Schalcher**	**2003**	**146**	**52±10**	**87**	**27±13**	**overall mortality + urgent transplant**	**61**	**41**	**18,4±5,4**	**14**	**no**
O' Neill*	2005	1196	54±11	75	19±7	overall mortality	72	nr	16,6±5,1	14	yes
**Bard**	**2006**	**355**	**51±10**	**72**	**22±8**	**overall mortality + urgent transplant**	**46**	**145**	**17,3±5,0**	**14**	**no**
**Nanas**	**2006**	**98**	**51±12**	**89**	**31±13**	**cardiac mortality**	**30**	**27**	**19,1±5,9**	**15**	**no**
**Guazzi**	**2007**	**288**	**55±13**	**67**	**33±13**	**cardiac mortality**	**33**	**62**	**15,5±5,0**	**14.1**	**no**
**Guazzi**	**2007**	**156**	**61±9,4**	**80**	**35±10**	**cardiac mortality**	**42**	**34**	**16,8±4,5**	**14.4**	**no**
Rossi	2007	273	62±9	87	33±8	cardiac mortality	75	40	16,6±4,5	16	yes
**Arena**	**2008**	**353**	**59±14**	**72**	**28±9**	**cardiac mortality + urgent transplant**	**48**	**104**	**14,5±5,6**	**14**	**no**
**Kazuhiro**	**2009**	**148**	**63±12**	**100**	**35±11**	**cardiac mortality**	**67**	**13**	**18,2±3,7**	**14**	**no**
**Arena**	**2010**	**520**	**58±12**	**77**	**35±14**	**cardiac mortality**	**48**	**79**	**16,6±6,2**	**14**	**no**
Sachdeva	2010	1215	53±13	75	23±7	overall mortality	24	234	13,1±2,0	14	yes

Bolded lines denote results with negative statistical significance.

**Table 4:** The 35 studies reporting a positive or negative statistical significance of a prognostic threshold of ejection fraction.

Author	Publication Year	Number of Patients	Age (years)	Males (%)	Primary Outcome	Max Duration of follow-up (months)	Number of Events	Mean ± SD EF(%)	Tested threshold (%)	Tested threshold prognostically significant?
Itoh	1992	298	63±11	57	overall mortality	40	167	35±nr	40	yes
Mehta	1992	112	66±8	74	cardiac mortality	78	31	33±nr	30	yes
Rihal	1994	102	61±14	63	overall mortality	36	35	23±8	25	yes
Omland	1995	145	61±10	80	overall mortality	44	36	51±10	49.1	yes
Andreas	1996	36	54±12	90	overall mortality	53	16	20±8	20	yes
Giannuzzi	1996	508	59±9	88	overall mortality + hospitalization	58	148	26±5	25	yes
Szabo	1997	159	61	85	overall mortality	69	30	26.6±9	27	yes
Anker	1997	171	60±11	90	overall mortality	18	49	30±15	25	yes
Wijbenga	1998	64	59±10	86	overall mortality + transplantation	30	64	31±8	30	yes
**Niebauer**	**1999**	**99**	**58±2**	**90**	**overall mortality**	**36**	**73**	**13,3±nr**	**10**	**no**
Metra	1999	219	55±10	93	overall mortality + urgent transplantation	144	38	22±7	20	yes
Isnard	2000	264	51±12	80	cardiac mortality + urgent transplantation	206	83	27±10	27	yes
Ghio	2000	140	52±11	75	cardiac mortality + urgent transplantation	38	52	22±2	20	yes
**McDonagh**	**2001**	**1640**	**50.4**	**48**	**overall mortality**	**48**	**80**	**47±nr**	**40**	**no**
**Neglia**	**2002**	**64**	**52±12**	**87**	**cardiac mortality**	**82**	**24**	**34±10**	**35**	**no**
Corrà	2002	600	58±7	88	cardiac mortality + urgent transplantation	102	87	26±4	25	yes
Szachniewicz	2003	176	63	86	overall mortality	18	32	42nr	35	yes
**Gardner**	**2003**	**142**	**50±10**	**82**	**cardiac mortality + urgent transplantation**	**55**	**24**	**14.9±7**	**13**	**no**
**Martinez-Selles**	**2003**	**1065**	**75**	**49**	**overall mortality**	**51**	**507**	**35±7**	**30**	**no**
**Shiba**	**2004**	**684**	**67±13**	**66**	**overall mortality**	**33**	**175**	**49±15**	**25**	**no**
Guazzi	2005	128	60±9	79	cardiac mortality	51	24	34±10	35	yes
Kistorp	2005	195	69	71	overall mortality	30	46	30±8	25	yes
Junger	2005	209	54±10	86	overall mortality	35	45	22±10	20	yes
**Peterson**	**2005**	**61**	**53±11**	**86**	**overall mortality + urgent transplantation**	**139**	**32**	**26±9**	**27**	**no**
**Bloomfield**	**2006**	**549**	**56±10**	**71**	**cardiac mortality**	**24**	**51**	**25±6**	**31**	**no**
Rossi	2007	273	62	87	overall mortality	45	44	31±3	30	yes
Arslan	2007	43	62±10	86	overall mortality	24	16	35±6	30	yes
Guazzi	2007	288	55±13	62	overall mortality	33	62	33±13	28	yes
vonHaeling	2007	525	61±12	94	overall mortality	28	171	28±4	20	yes
**Nishio**	**2007**	**145**	**67±1.8**	**70**	**cardiac mortality + hospitalization**	**33**	**28**	**31±nr**	**30**	**no**
**Dini**	**2007**	**356**	**70±6**	**22**	**cardiac death**	**34**	**54**	**31±3**	**25**	**no**
**Whalley**	**2008**	**228**	**70±3**	**66**	**cardiac mortality + hospitalization**	18	**26**	**57±12**	**45**	**no**
**Dini**	**2008**	**142**	**71±11**	**78**	**overall mortality**	**50**	**85**	**28±7**	**25**	**no**
**Parissis**	**2009**	**300**	**65±11**	**83**	**cardiac mortality + hospitalization**	**12**	**92**	**28±4**	**25**	**no**
Smilde	2009	90	60±8	85	overall mortality	156	47	29±9	30	yes

Bolded lines denote results with negative statistical significance.

**Table 5:** The 20 studies reporting a positive or negative statistical significance of a prognostic threshold of brain natriuretic peptide.

Author	Publication Year	Number of patients	Age (years)	Males (%)	Primary Outcome	Max duration of follow-up (months)	Number of events	Median (IQR) BNP (ng/L)	Tested threshold (ng/L)	Tested threshold prognostically significant
Omland	1996	131	68±1	75	overall mortality	48	31	33.1	33.3	yes
Yu	1999	91	61	70	cardiac mortality	12	25	165	165	yes
Bettencourt	2004	84	69±9	60	overall mortality	nr	17	260,4 (122,4-543,8)	260.4	yes
de Groote	2004	150	55±13	nr	cardiac mortality	24	35	107 (3,5-876)	260	yes
Hulsmann	2005	112	68±12	64	overall mortality	43	nr	231	231	yes
Watanabe	2005	417	64±14	69	overall mortality + hospitalization	nr	124	132	81	yes
Lamblin	2005	546	56	82	cardiac mortality + urgent trasplantantion	53	113	173	173	yes
Bertinchant	2005	63	54±7.2	89	cardiac mortality + hospitalization	nr	47	89,5 (11-1413)	254	yes
Horwich	2006	316	53±13	74	overall mortality	48	nr	452	452	yes
Masson	2006	3916	nr	nr	overall mortality	nr	758	99	125	yes
Sun	2007	50	67±6	58	cardiac mortality	24	12	780	520	yes
Frantz	2007	206	60±	80	overall mortality + hospitalization	12	81	141	141	yes
Christ	2007	123	63±12	85	overall mortality + urgent trasplantantion	36	28	183 (11-1672)	183	yes
Dhaliwal	2009	464	67±7	99	overall mortality + hospitalization	nr	126	490 (233-796)	350	yes
Moertl	2009	96	69±12	58	overall mortality + hospitalization	24	34	267	267	yes
Niessner	2009	351	75±nr	66	overall mortality + hospitalization	16	175	441 (231-842)	441	yes
Cohen-Solal	2009	1038	66	70	overall mortality	6	nr	768	800	yes
El-Saed	2009	173	67±11	98	overall mortality	24	31	315	492	yes
**Voors**	**2009**	**224**	**68±10**	**70**	**overall mortality + resuscitated arrest**	**12**	**63**	**109**	**181**	**no**
Sachdeva	2010	1215	53±13	75	overall mortality + urgent trasplantantion	24	442	575 (190-1300)	579	yes

Bolded lines denote results with negative statistical significance.

The images for Figures 6 and 7 are incorrectly switched. The image that appears as Figure 6 should be Figure 7, and the image that appears as Figure 7 should be Figure 6. The figure legends appear in the correct order. The correct Figure 6 and Figure 7 can be viewed below.

**Figure 6:**
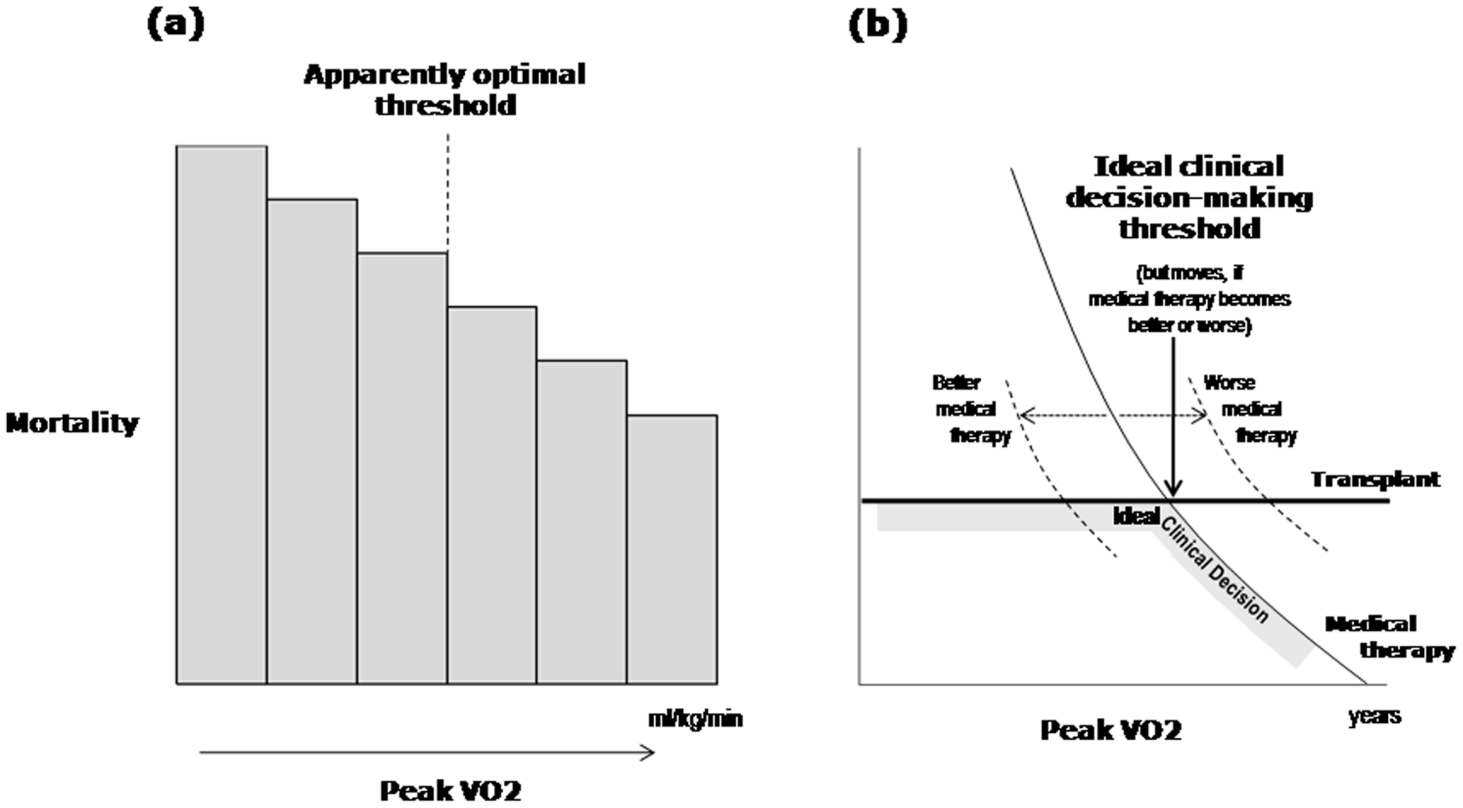
Two different types of threshold: apparently-optimal versus decision-making thresholds. Cartoon illustrating two distinct, unrelated, values that are both called “threshold”. The statistically optimal threshold value of a continuous risk factor for subdividing the population (left panel) has no relevance to the question of what value of a risk factor should be used to decide whether to intervene or not (right panel). The former, the “observed prognostic threshold”, will generally be the middle of whatever population happens to be studied, if mortality varies roughly linearly with the risk factor. The latter, the “ideal clinical decision-making threshold”, will critically depend also on the outcomes with intervention, and will move as the success of the package of medical therapy (and of transplantation) changes with time. There is no sense in using one as a proxy for the other.

**Figure 7:**
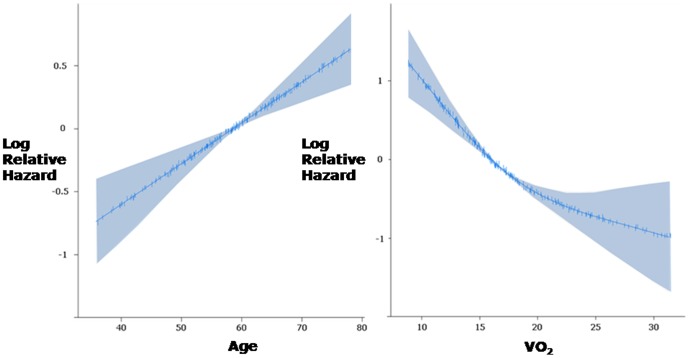
Example of use of flexible non-linear function to describe the relationships between age (left) and peak VO_2_ (right) and log odds of death using 208 patients. The shaded areas represent the 95% confidence intervals for this function. Flexible non-linear functions have numerous benefits over categorization, including improved precision, avoidance of assumption of a discontinuous relationship, maximisation of applicability to the individual and importantly avoidance of giving other variables or interactions artificially high weights. Inspection of the resulting plots above can make obvious the lack of a discontinuity in risk.

In the Discussion section, the third paragraph under the title “Two easily-confused but different types of ‘threshold,’” the first sentence states “That these two types of threshold differ is sketched in Figure 5, which imagines a situation where, with only medical therapy, mortality falls smoothly with rising peak VO_2_, while with transplantation mortality is at a fixed level.” This should refer to Figure 6, which can be seen below.

Also in the Discussion section, under “Prognostic studies,” the second sentence of the first paragraph states “For example, a flexible nonlinear function can be fitted and displayed with confidence bands for incremental log odds over the whole span of the marker; seeking a point such that risk is flat on both sides of that point but the risk on one side is much different from the risk on the other side (Figure 6).” This should refer to Figure 7, which can be seen below.
