# Supplementary material for: Do Optimal Prognostic Thresholds in Continuous Physiological Variables Really Exist? Analysis of Origin of Apparent Thresholds, with Systematic Review for Peak Oxygen Consumption, Ejection Fraction and BNP
Source: PLoS One. 2014 Jan 27;9(1):e81699. doi: 10.1371/journal.pone.0081699 (PMC3903471; doi:10.1371/journal.pone.0081699)
Supplement: Checklist S1 — PRISMA checklist. (DOC) [file pone.0081699.s001.doc]

Text S1 - Checklist of items to include when reporting a systematic review or meta-analysis

| Section/topic | # | Checklist item | Reported on page # |
| --- | --- | --- | --- |
| **TITLE** | | | |
| Title | 1 | Identify the report as a systematic review, meta-analysis, or both. | **Title**, page 1 |
| **ABSTRACT** | | | |
| Structured summary | 2 | Provide a structured summary including, as applicable: background; objectives; data sources; study eligibility criteria, participants, and interventions; study appraisal and synthesis methods; results; limitations; conclusions and implications of key findings; systematic review registration number. | **Abstract**, pages 2 and 3  The applicable headings are now present |
| **INTRODUCTION** | | | |
| Rationale | 3 | Describe the rationale for the review in the context of what is already known. | **Introduction**, page 4, first paragraph and last sentence of the second paragraph |
| Objectives | 4 | Provide an explicit statement of questions being addressed with reference to participants, interventions, comparisons, outcomes, and study design (PICOS). | **Introduction**, page 5, last paragraph |
| **METHODS** | | | |
| Protocol and registration | 5 | Indicate if a review protocol exists, if and where it can be accessed (e.g., Web address), and, if available, provide registration information including registration number. | This is a special study combining a systematic review and a mathematical simulation. There is no registration number. |
| Eligibility criteria | 6 | Specify study characteristics (e.g., PICOS, length of follow-up) and report characteristics (e.g., years considered, language, publication status) used as criteria for eligibility, giving rationale. | **Methods**, Part 1, page 6:  - Examination of Published Studies  - Selection criteria |
| Information sources | 7 | Describe all information sources (e.g., databases with dates of coverage, contact with study authors to identify additional studies) in the search and date last searched. | **Methods**, Part 1, page 6:  - Examination of Published Studies  - Selection criteria |
| Search | 8 | Present full electronic search strategy for at least one database, including any limits used, such that it could be repeated. | **Methods**, Part 1, page 6:  - Examination of Published Studies  - Selection criteria |
| Study selection | 9 | State the process for selecting studies (i.e., screening, eligibility, included in systematic review, and, if applicable, included in the meta-analysis). | **Methods**, Part 1, page 6:  - Examination of Published Studies  - Selection criteria |
| Data collection process | 10 | Describe method of data extraction from reports (e.g., piloted forms, independently, in duplicate) and any processes for obtaining and confirming data from investigators. | **Methods**, Part 1, page 6: Examination of Published Studies |
| Data items | 11 | List and define all variables for which data were sought (e.g., PICOS, funding sources) and any assumptions and simplifications made. | **Methods**, Part 1, page 6:  - Examination of Published Studies  and  - Selection criteria  **Tables** 1, 4 and 5, pages 27, 30 and 31 |
| Risk of bias in individual studies | 12 | Describe methods used for assessing risk of bias of individual studies (including specification of whether this was done at the study or outcome level), and how this information is to be used in any data synthesis. | This is not a study of effect size, but of the relationship between the apparently-optimal prognostic thresholds found and the middle of the sample.  Our whole study is an exploration of a type of bias, which is extensively explained in the discussion, pages 15-22. |
| Summary measures | 13 | State the principal summary measures (e.g., risk ratio, difference in means). | Not applicable: this is not a study of effect size of an intervention. |
| Synthesis of results | 14 | Describe the methods of handling data and combining results of studies, if done, including measures of consistency (e.g., I^2^) for each meta-analysis. | Not applicable, this is not a meta-analysis. |
| Risk of bias across studies | 15 | Specify any assessment of risk of bias that may affect the cumulative evidence (e.g., publication bias, selective reporting within studies). | See item 12. |
| Additional analyses | 16 | Describe methods of additional analyses (e.g., sensitivity or subgroup analyses, meta-regression), if done, indicating which were pre-specified. | Not performed. |
| RESULTS | | | |
| Study selection | 17 | Give numbers of studies screened, assessed for eligibility, and included in the review, with reasons for exclusions at each stage, ideally with a flow diagram. | **Methods**, Part 1, page 6: - Examination of Published Studies  **Results,** page 10:  - Peak VO2 thresholds in published data, first paragraph. |
| Study characteristics | 18 | For each study, present characteristics for which data were extracted (e.g., study size, PICOS, follow-up period) and provide the citations. | **Methods**, Part 1, page 6:  - Examination of Published Studies  - Selection criteria  **Results**, pages 10-12:  - Peak VO2 thresholds in published data, first paragraph.  - Published thresholds in ejection fraction,  first paragraph.  - Published thresholds in Brain Natriuretic Peptide, first paragraph  **Tables** 1, 4 and 5,  pages 27, 30 and 31. |
| Risk of bias within studies | 19 | Present data on risk of bias of each study and, if available, any outcome-level assessment (see Item 12). | See Item 12. |
| Results of individual studies | 20 | For all outcomes considered (benefits or harms), present, for each study: (a) simple summary data for each intervention group and (b) effect estimates and confidence intervals, ideally with a forest plot. | **Table** 1, 4 and 5,  pages 27, 30 and 31. |
| Synthesis of results | 21 | Present results of each meta-analysis done, including confidence intervals and measures of consistency. | The study is not a meta-analysis. |
| Risk of bias across studies | 22 | Present results of any assessment of risk of bias across studies (see Item 15). | See Item 12. |
| Additional analysis | 23 | Give results of additional analyses, if done (e.g., sensitivity or subgroup analyses, meta-regression [see Item 16]). | Not performed. |
| DISCUSSION | | | |
| Summary of evidence | 24 | Summarize the main findings including the strength of evidence for each main outcome; consider their relevance to key groups (e.g., health care providers, users, and policy makers). | **Discussion**, pages 15, 16-7 and 20:  - First paragraph  - Lessons learnt from peak VO2, EF, and BNP studies.  - Clinical implication. |
| Limitations | 25 | Discuss limitations at study and outcome level (e.g., risk of bias), and at review level (e.g., incomplete retrieval of identified research, reporting bias). | **Discussion**, page 21:  **-** Study limitations section. |
| Conclusions | 26 | Provide a general interpretation of the results in the context of other evidence, and implications for future research. | **Conclusions** |
| FUNDING | | | |
| Funding | 27 | Describe sources of funding for the systematic review and other support (e.g., supply of data); role of funders for the systematic review. | Funding Sources provided to the journal. |
